# Microbiome-Associated Regulation of Isoquinoline Alkaloids in *Sinomenium acutum* Reveals Functional Endophytes for Sinomenine Accumulation

**DOI:** 10.3390/plants15203211

**Published:** 2026-10-19

**Authors:** Ziyi Xiao, Sili Yin, Yulu Zhang, Wendi Lan, Le Huang, Ye Yuan, Xiyao Zeng, Weijun Zeng, Zhaojing Yu, Wenbin Xiao, Yichen Tang, Hua Yang, Yunhua Xiao

**Affiliations:** 1 College of Bioscience and Biotechnology, Hunan Agricultural University, Changsha 410128, China; 2 Yuelushan Laboratory, Changsha 410128, China; 3 Hunan Zhengqing Pharmaceutical Group Co., Ltd., Huaihua 418000, China

**Keywords:** *Sinomenium acutum*–endophyte interactions, *Microbacterium* sp., *Cladosporium* sp., bioactive compounds, functional inoculation

## Abstract

*Sinomenium acutum* produces isoquinoline alkaloids such as sinomenine, but the role of endophytes in alkaloid biosynthesis and accumulation remains unclear. This study integrated 16S/ITS amplicon sequencing, untargeted metabolomics, and culture-dependent strain isolation to compare endophytic community structures and metabolite profiles between *S. acutum* roots with high (HC) and low (LC) isoquinoline alkaloid contents. Compared with the LC group, the HC group showed significantly higher levels of amino acids and their derivatives (e.g., Tyr-Trp), as well as isoquinoline alkaloids. A total of 49 bacterial strains and 20 fungal strains were isolated. Spearman’s correlation analysis revealed that two *Microbacterium* amplicon sequence variants (ASVs) were positively correlated with the contents of total tyrosine derivatives and sinomenine, while *Cladosporium* ASV40 was positively correlated with the content of sinoacutine, a precursor of sinomenine. Targeted LC-MS/MS analysis tentatively annotated isoquinoline and 8-hydroxyquinoline in G4 cultures and sinomenine in P37 cultures (MSI level 2). Importantly, in a six-month field trial, foliar spraying of *Microbacterium* sp. G4 significantly increased sinomenine content but significantly decreased (*S*)-reticuline content in *S. acutum* roots—consistent with enhanced channeling of this branch-point precursor toward sinomenine–supporting a functional association between G4 inoculation and sinomenine accumulation. These findings reveal previously unknown endophyte–metabolite associations in *S. acutum* and provide a basis for microbe-assisted improvement of the medicinal quality of *S. acutum*.

## 1. Introduction

*Sinomenium acutum* (Thunb.) Rehd. et Wils., a traditional Chinese medicinal plant utilized for over 1000 years, is predominantly distributed in Japan, Vietnam, and China. *S. acutum* is renowned for its rich spectrum of secondary metabolites, particularly alkaloids and terpenoids, which are an important source of bioactive agents [1]. In traditional Chinese medicine theory, *S. acutum* is characterized by bitter and pungent flavors and is used to treat rheumatoid arthritis and alleviate edema [2,3]. Modern pharmacological research has further substantiated its immunomodulatory, anticancer and antimicrobial activities [4,5].

The principal bioactive constituents of *S. acutum* are isoquinoline alkaloids, including sinomenine, sinoacutine, (*S*)-reticuline, and (*S*)-scoulerine. These compounds exert potent immunomodulatory effects, notably through selective inhibition of pro-inflammatory signaling pathways such as JAK/STAT and NF-κB [6]. Consequently, preparations derived from *S. acutum*, particularly its dried roots rich in sinomenine, have been developed into commercial drugs (e.g., Zhengqing Fengtongning injection and enteric-coated tablets) [7,8]. However, indiscriminate and excessive collection has severely depleted wild *S. acutum* resources, and the alkaloid content of artificially cultivated *S. acutum* remains unstable, posing a major constraint to commercial production [9,10].

Endophytic microorganisms, which reside within plant tissues without causing disease, can produce active metabolites and modulate host secondary metabolism [11]. In some medicinal plants, endophytes even synthesize bioactive compounds identical or similar to those of the host [12]. These symbionts influence medicinal compound biosynthesis through multiple mechanisms: provision of precursors, horizontal transfer of biosynthetic genes (e.g., P450 enzymes), and de novo synthesis of structural analogs [13,14]. For instance, endophytes isolated from *Catharanthus roseus* produced vinblastine independently [15], and *Taxus*-associated microbes yield paclitaxel congeners with antitumor activity [16]. Collectively, these findings underscore the potential of plant endophytic microbial metabolites as alternative sources of bioactive compounds otherwise obtained from endangered medicinal species.

Recently, our research group has focused on the roles of endophytes in the biosynthesis of the active compounds in *S. acutum*. Yin et al. [17] employed an iterative enrichment cultivation strategy combined with multiomic analysis to identify endophytic bacteria potentially affecting sinomenine synthesis and successfully isolated a *Priestia aryabhattai* strain capable of independently producing sinomenine. Xiao et al. [18] compared the endophytic community compositions across different tissues of male and female *S. acutum*, providing a baseline understanding of tissue-specific and sex-specific microbial patterns. These findings collectively demonstrated that endophytes in *S. acutum* are functionally relevant to alkaloid biosynthesis.

Despite these advances, it remains unclear whether plants with high and low alkaloid contents harbor distinct core endophytic populations, and whether these populations contribute to the observed differences. To address this gap, the present study investigated the functional relationships between root endophytic communities and isoquinoline alkaloid accumulation in *S. acutum*. By integrating community profiling, metabolomics, strain isolation, fermentation assays, and field inoculation experiments, this study provides a comprehensive view of how endophytic microbes modulate alkaloid biosynthesis in *S. acutum*, offering a sustainable strategy for conserving wild resources and improving the contents of active compounds.

## 2. Materials and Methods

### 2.1. Plant Sample Collection and Grouping

The sampling site was located at the *S. acutum* cultivation base along National Highway 209 in Zhongfang County, Huaihua City, Hunan Province, China (109.91° E, 27.38° N). To minimize the influence of confounding variables such as plant age, microenvironment, soil microhabitat, and sampling time, all root samples were collected from healthy, four-year-old male *S. acutum* plants growing within a geographically confined and visually homogeneous cultivation area during a single sampling event on 15 October 2023. A total of 12 individual plants were sampled. Each root was carefully freed of adhering soil, rinsed thoroughly with ultrapure water, and placed into sterile polyethylene bags. The root material from each plant was then divided into four portions. The first portion was flash-frozen in liquid nitrogen and stored on dry ice for isoquinoline alkaloid quantification (including sinomenine); the second portion was similarly processed for untargeted metabolomics analysis; the remaining two portions were surface-sterilized with 75% ethanol, rinsed with sterile distilled water, and used for endophytic microbiome sequencing and endophyte isolation, respectively. The first two portions were maintained on dry ice, whereas the latter two were stored at 4 °C; all samples were transported to the laboratory and processed within 24 h.

### 2.2. Determination of Sinomenine, Sinoacutine, and (S)-Reticuline by HPLC

The contents of sinomenine, sinoacutine, and (*S*)-reticuline were determined by high-performance liquid chromatography (HPLC, Waters 2694, Milford, MA, USA) following the method of Zhou et al. [19] with modifications. Reference standards of sinomenine, sinoacutine, and (*S*)-reticuline (Shanghai Chemsoon Biotechnology Co., Ltd., Shanghai, China) were used to prepare stock solutions and construct calibration curves over the concentration ranges listed in Figure S1. The dried root samples were ground into a fine powder. An accurately weighed 0.2 g portion of each sample was transferred into a 2 mL volumetric flask. For the extraction of sinomenine and sinoacutine, 70% ethanol was used as the solvent; for (*S*)-reticuline, a methanol–hydrochloric acid solution (100:1, *v*/*v*) was employed. Each sample was sonicated (180 W, 59 kHz) for 20 min, cooled to room temperature, and made up to volume. The mixture was thoroughly shaken and filtered through a 0.22 µm microporous membrane, and the filtrate was collected as the test solution. Chromatographic separation was performed on an Agilent C18 column (Agilent Technologies, Santa Clara, CA, USA, dimensions: 4.6 mm × 150 mm, 5 µm). The mobile phases consisted of methanol (phase A) and phosphate buffer (phase B; 0.005 mol/L sodium phosphate mixed with 0.005 mol/L sodium dihydrogen phosphate to pH 8.0, then adjusted to pH 9.0 with 1% triethylamine aqueous solution). Isocratic elution was performed with A:B = 45:55 (*v*/*v*) at a flow rate of 1 mL/min; the column temperature was 24 °C, the injection volume was 10 µL, and the detection wavelength was set at 262 nm.

Based on the HPLC-quantified alkaloid contents, the 12 individuals were ranked according to sinomenine content: the six plants with the higher contents (42.73–53.66 mg/g) were assigned to the high-content (HC) group, whereas the six with the lower contents (27.86–40.66 mg/g) formed the low-content (LC) group, with no overlap between the two groups. This pre-grouping was subsequently confirmed by principal component analysis (PCA) of both the metabolomic and microbiome datasets (Section 3.1 and Section 3.2).

### 2.3. Non-Targeted Metabolomics Analysis

Sample preparation, extract analysis, metabolite identification, and quantification were performed according to a previously published protocol [18]. Metabolite profiling was conducted by Metware Biotechnology Co., Ltd. (Wuhan, China). Briefly, root samples were vacuum freeze-dried (Scientz-100F, Scientz-100F, Ningbo Scientz Biotechnology Co., Ltd., Ningbo, China) and ground to a fine powder (30 Hz, 1.5 min; MM 400, Retsch GmbH, Haan, Germany). A 50 mg portion of each sample was weighed on an electronic balance (MS105DU, Mettler Toledo, Greifensee, Switzerland) and extracted with 1200 µL of −20 °C pre-cooled 70% methanol containing the internal standard (2-chloro-L-phenylalanine; Metware Biotechnology Co., Ltd., Wuhan, China); the mixture was vortexed for 30 s every 30 min (six times in total) and centrifuged at 12,000 rpm for 3 min, and the supernatant was filtered through a 0.22 μm microporous membrane and transferred to an injection vial for UPLC-QTOF/MS analysis. Chromatographic separation was performed on a Waters ACQUITY UPLC HSS T3 column (1.8 µm, 2.1 × 100 mm) at 40 °C, with a flow rate of 0.40 mL/min and an injection volume of 4 µL; the mobile phase consisted of water containing 0.1% formic acid (phase A) and acetonitrile containing 0.1% formic acid (phase B), with the following elution gradient: 0 min, 95% A; 5 min, 35% A; 6 min, 1% A, held for 1.5 min; 7.6 min, 95% A, held for 2.4 min. Mass spectrometric data were acquired on a TripleTOF 6600+ mass spectrometer (AB SCIEX, Concord, ON, Canada) coupled to a Waters UHPLC system and operated in information-dependent acquisition (IDA) mode using Analyst TF 1.7.1 software (SCIEX). The source parameters were as follows: ion source gases 1 and 2, 50 and 60 psi, respectively; curtain gas, 35 psi; source temperature, 550 °C; declustering potential, +80 or −80 V; and ion spray voltage floating, +5500 or −4500 V in positive and negative ion modes, respectively. TOF MS scans covered *m*/*z* 50–1250 with an accumulation time of 200 ms and dynamic background subtraction enabled; product ion scans covered *m*/*z* 50–1250 with an accumulation time of 40 ms, a collision energy of ±30 V, and a collision energy spread of 15 V. Metabolic data were analyzed using the Metware Cloud Platform (https://cloud.metware.cn). Raw UPLC-QTOF/MS data were processed on the platform with peak detection, retention-time alignment, and peak-area normalization; features present in the extraction blanks were removed (blank subtraction), and all differential features were manually inspected for chromatographic peak shape and integration quality. Differential metabolites between the two groups were identified by orthogonal projections to latent structures–discriminant analysis (OPLS-DA). Metabolites with VIP ≥ 1 were selected, and Student’s *t*-tests were performed for each metabolite; the resulting *p*-values were corrected using the Benjamini–Hochberg method. Metabolites were considered significantly altered if they presented an adjusted *p*-value < 0.05 (FDR-corrected) and an absolute |log_2_(fold change)| ≥ 1. Metabolite annotation confidence was assigned according to the minimum reporting standards of the Metabolomics Standards Initiative (MSI) [20]: level 1, confirmed structures based on at least two orthogonal properties (e.g., retention time and MS/MS spectrum) matching an authentic reference standard analyzed under identical experimental conditions; level 2, putatively annotated compounds based on MS/MS spectral similarity with public or in-house libraries in the absence of authentic standards; and level 3, putatively characterized compound classes. In this study, no authentic standards were co-analyzed for the untargeted dataset; accordingly, all annotations are reported at MSI level 2 at best.

### 2.4. DNA Extraction, PCR Amplification, and Illumina HiSeq Sequencing

Before total DNA extraction, the roots of *S. acutum* were subjected to surface disinfection according to Deng et al. [21] to effectively remove epiphytic microorganisms. Total DNA was then extracted using the TIANamp Stool DNA Kit (Cat#DP328, TIANGEN Biotech Co., Ltd., Beijing, China) following the manufacturer’s instructions. Throughout the DNA extraction and subsequent PCR amplification, stringent laboratory practices were followed to minimize potential environmental and reagent contamination, including the use of sterile, DNA-free consumables and reagents. To monitor contamination, extraction blanks (no sample) were processed in parallel, and no-template controls (NTC) were included in each PCR run. The V5-V7 region of the bacterial 16S rRNA gene (799F/1193R) and the fungal ITS1 region (ITS1-1F-F/ITS1-1F-R) were amplified. PCR was performed with an annealing temperature of 50 °C for 30 cycles, as described by Gao et al. [22]; full reaction conditions are provided in Supplementary Materials. No detectable amplification was observed in the NTC or extraction blank controls, confirming the absence of significant contamination. Amplicon libraries were sequenced on the Illumina HiSeq 2500 platform (performed by Novogene Co., Ltd., Guangzhou, China).

The bacterial 16S rRNA gene and fungal ITS sequences were processed using QIIME 2 (v2022.2). Denoising and chimera removal were performed with DADA2 to obtain non-chimeric amplicon sequence variants (ASVs) [23]. Taxonomic assignment was performed using the SILVA 138 database for bacterial 16S rRNA gene sequences and the UNITE (v9.0) database for fungal ITS sequences. Sequences annotated as chloroplast, mitochondria, or unassigned at the kingdom level were removed before downstream analyses. For diversity analyses, samples were rarefied to an even sequencing depth. The raw sequencing data have been deposited in the NCBI SRA database under accession numbers PRJNA1287093 and PRJNA1287097.

Venn diagrams were used to visualize shared and unique ASVs between the groups. PCA was utilized to analyze differences in microbial community composition among the samples. Shannon and Pielou indices were calculated to assess the alpha diversity and evenness of species among the samples, and Chao1 and ACE indices were calculated to assess richness. Bubble plots of relative abundance were used to analyze species composition and highlight core microorganisms, and histograms were used to depict the relative abundance of microorganisms and identify dominant taxa within different groups.

### 2.5. Isolation, Purification, and Identification of Endophytes from S. acutum

Fresh *S. acutum* roots were collected for endophyte isolation, purification, and identification following Khalil et al. [24], with the modification that surface sterilization was performed with 75% ethanol for 5 min, followed by 4% sodium hypochlorite for 10 min. Surface-sterilized roots were cut into small pieces (approximately 0.5 × 0.5 cm) and inoculated onto three different media: LB (Luria–Bertani), Gauze’s Synthetic Medium No.1, and PDA (Potato Dextrose Agar). To verify the efficacy of surface sterilization, 100 μL of the final rinse water was spread onto each medium and incubated under the same conditions; only plates showing no microbial growth were considered valid [24]. Plates were incubated at 30 °C for 3–7 days (bacteria) and at 25 °C for 5–14 days (fungi). All potentially different colonies were transferred to separate plates with a sterile needle for further purification [25]. Individual colonies were preserved in cryogenic tubes in 30% glycerol at −80 °C for further study [26,27].

For the endophytic bacteria, the 16S rRNA gene was amplified with primers 27F (AGAGTTTGATCCTGGGTCAG) and 1492R (GGTTACCTTGTTACGACTT) using total DNA as the template. For the endophytic fungi, the ITS rDNA was amplified with primers ITS1 (TCCGTAGGTGAACCTGCGG) and ITS4 (TCCTCCGCTTATTGATATGC). Sequencing was performed by Sangon Biotech Co., Ltd. (Shanghai, China). The assembled sequences were compared against the NCBI databases using BLASTn (https://blast.ncbi.nlm.nih.gov/Blast.cgi, accessed on 4 March 2025), and sequences exhibiting >99% similarity were selected for phylogenetic tree construction. Phylogenetic and molecular evolutionary analyses were conducted using the Neighbor-Joining method with 1000 bootstrap replicates in MEGA X [28]. Morphological characterization of mycelial or cellular morphology was performed using optical microscopy.

### 2.6. Morphological Observation of Key Microorganisms

To assess cellular and mycelial ultrastructure, samples were fixed with 2.5% glutaraldehyde at 4 °C for 2 h, rinsed with phosphate-buffered saline (PBS; pH 7.2), and dehydrated through a graded ethanol series (10%, 30%, 50%, 70%, 90%, and 100%, 15 min each). The samples were then treated twice with 100% ethanol for 20 min, followed by two exchanges with tert-butanol (20 min each). Subsequently, the samples were subjected to vacuum freeze-drying, gold sputter-coating, and examination by scanning electron microscopy (SEM; Hitachi SU8010, Hitachi High-Tech, Tokyo, Japan).

### 2.7. Liquid Fermentation of Key Microorganisms

*Microbacterium* sp. G4 was fermented in Czapek-Dox medium (composition per liter: NaNO_3_ 3 g, K_2_HPO_4_ 1 g, MgSO_4_ 0.5 g, KCl 0.5 g, FeSO_4_ 0.01 g, sucrose 30 g; pH 7.0–7.2; made up to 1 L with distilled water) at 30 °C and 160 rpm for 3 days. Two treatments were established: SG4, in which the medium was supplemented with 1.7 g/L L-tyrosine, and FG4, in which the same medium was used without L-tyrosine. Each treatment comprised three biological replicates, with uninoculated blank medium serving as the negative control. After fermentation, the whole culture broth was sonicated for 5 min and centrifuged at 12,000 rpm and 4 °C for 10 min, and the supernatant was collected for targeted alkaloid analysis as detailed in Section 2.8.

*Cladosporium* sp. P37 was cultured in 100 mL of PDB liquid medium (Qingdao Hope Biotechnology Co., Ltd., Qingdao, China) inoculated with a 5 mm-diameter mycelial agar plug and incubated at 25 °C with shaking at 160 rpm for 10 days. After ultrasonication and centrifugation as described above, the supernatant pH was adjusted to 9–11 with concentrated ammonia water and extracted three times with an equal volume of dichloromethane. The combined organic phases were evaporated to dryness under a gentle nitrogen stream, and the residue was reconstituted in a defined volume of 70% methanol for alkaloid-targeted analysis as detailed in Section 2.8. Each treatment comprised three biological replicates, with uninoculated blank medium serving as the negative control.

### 2.8. Alkaloid-Targeted Metabolism of Fermentation Products and Specific MS Parameters

All sample extracts were subjected to a uniform preparation protocol. An aliquot (200 µL) was mixed with 200 µL of ice-cold 70% methanol containing 1 mg/L 2-chloro-L-phenylalanine (98% purity) as an internal standard for monitoring extraction and instrument stability. The mixture was vortexed for 15 min, centrifuged at 12,000 rpm for 3 min at 4 °C, and the supernatant was filtered through a 0.22 μm microporous membrane for UPLC-MS/MS analysis.

The analysis was performed on the UPLC ExionLC™ AD system coupled to a QTRAP 4500 mass spectrometer (SCIEX, Framingham, MA, USA). Chromatographic separation was achieved on an Agilent SB-C18 column (1.8 µm, 2.1 mm × 100 mm) maintained at 40 °C. Mobile phase A was ultrapure water with 0.1% formic acid, and mobile phase B was acetonitrile with 0.1% formic acid. The gradient program was as follows: 0 min, 5% B; 9 min, 95% B; 10 min, 95% B; 11.1 min, 5% B; 14 min, 5% B. The flow rate was 0.35 mL/min, and the injection volume was 2 μL.

MS detection was conducted in electrospray ionization (ESI) positive mode with the following parameters: source temperature 500 °C; ion spray voltage 5500 V; ion source gas I (GSI), 50 psi; gas II (GSII), 60 psi; curtain gas (CUR), 25 psi; collision-activated dissociation (CAD) gas, medium. Multiple reaction monitoring (MRM) was employed for quantification. The MRM transitions, declustering potential (DP), and collision energy (CE) for each targeted alkaloid were optimized with standard solutions; the detailed MRM parameters were provided in Table S3. Quality control (QC) samples were prepared and injected throughout the analytical run to monitor signal drift and instrument stability, with one QC sample analyzed every six samples; the QC injections were used for monitoring only, and no drift correction was applied. Metabolite annotation was based on the concordance of retention time and MS/MS fragmentation patterns (Q1/Q3 MRM transitions) with an in-house library (RT tolerance 0.2 min). Because the QTRAP 4500 is a unit-resolution triple-quadrupole instrument, no accurate-mass information was used for annotation. Annotation confidence was assigned following the MSI criteria described in Section 2.3. Because no authentic standards were co-analyzed under identical conditions, isoquinoline and sinomenine detected in the fermentation cultures are reported as putatively annotated compounds (MSI level 2).

### 2.9. Field Inoculation Trial with Microbacterium sp. G4

The field-inoculation protocol and the field results for strain P37 were reported in our companion study [18]; the fermentation experiments with P37 presented in Section 3.5 were new experiments performed in parallel with those of G4 in the present study. The field trial was conducted at the *S. acutum* cultivation base along National Highway 209 in Zhongfang County, Huaihua City, Hunan Province, China (109.91° E, 27.38° N), from 9 July 2025 to 8 January 2026. Healthy one-year-old *S. acutum* plants were randomly assigned to three treatments (foliar spraying, root irrigation, and a sterile-water control, CK), with 30 plants per treatment, from which 6 plants per group were randomly selected for alkaloid quantification. The three treatments were arranged in a single contiguous field block following a completely randomized design, with treatment assignment by random number table. All plants received uniform standard cultivation management, and no fertilizers or pesticides were applied during the trial. Baseline alkaloid contents were not measured, which we acknowledged as a limitation. During the trial period, the mean daily temperature ranged from −2 to 38 °C. Colonization of G4 in the inoculated plants was not verified (see Section 3.6). Inoculations were performed monthly during the first three months and every two months thereafter over the six-month experimental period. Sample collection and alkaloid quantification were performed following the methods described for P37 in our companion study [18], with minor modifications to the cultivation and pretreatment procedures. Specifically, the activated G4 strain was inoculated into LB liquid medium (10 g/L tryptone, 5 g/L yeast extract, 10 g/L NaCl) and incubated with shaking at 30 °C and 150 rpm for 3 days until the medium became noticeably turbid. Under aseptic conditions, the bacterial suspension was centrifuged at 8000 rpm for 10 min, the supernatant was discarded, and the cells were resuspended in sterile water; the bacterial concentration was adjusted to 1 × 10^7^ CFU/mL using the plate-count method. The contents of three isoquinoline alkaloids (sinomenine, (*S*)-coclaurine, and (*S*)-reticuline) in the roots were quantified by HPLC following the method described by Xiao et al. [18]. For each application, 300 mL of the G4 suspension was applied per plant, either by foliar spraying or by root irrigation, and CK plants received an equal volume of sterile water.

### 2.10. Statistical Analysis

For two-group comparisons (HC vs. LC) and three-group comparisons (field inoculation trial), one-way ANOVA followed by Tukey’s post hoc test was used. Statistical analysis was performed in Minitab 16 (Minitab, LLC, State College, PA, USA), and figures were plotted using GraphPad Prism 8 (GraphPad Software, San Diego, CA, USA). Data were confirmed to be approximately normally distributed (Shapiro–Wilk test, *p* > 0.05).

To elucidate the interplay between root metabolites and microorganisms, molecular ecological networks were constructed using a co-occurrence model based on ASV-level abundances of endophytic microbes and untargeted metabolomics data. Intergroup networks were constructed on the Tutools Cloud platform (https://www.cloudtutu.com, accessed on 4 March 2025) using Spearman’s rank correlations (|*R*| > 0.6, *q* < 0.05). Based on these networks, ASVs of key microorganisms significantly associated with isoquinoline alkaloids and L-tyrosine derivatives were selected for subnetwork construction under the same thresholds. The constructed networks were validated against 10,000 degree-preserving randomized networks in R 4.3.2 (R Foundation for Statistical Computing, Vienna, Austria) by comparing their topological characteristics. In addition, Spearman’s correlation heatmaps between the ASVs corresponding to the isolated endophytic genera, the HPLC-quantified isoquinoline alkaloid contents, total L-tyrosine derivatives, and other high-content isoquinoline alkaloids from the untargeted metabolome were constructed on the same platform.

## 3. Results

### 3.1. Quantification of Major Isoquinoline Alkaloids and Non-Targeted Metabolomics Analysis

**Figure 1:**
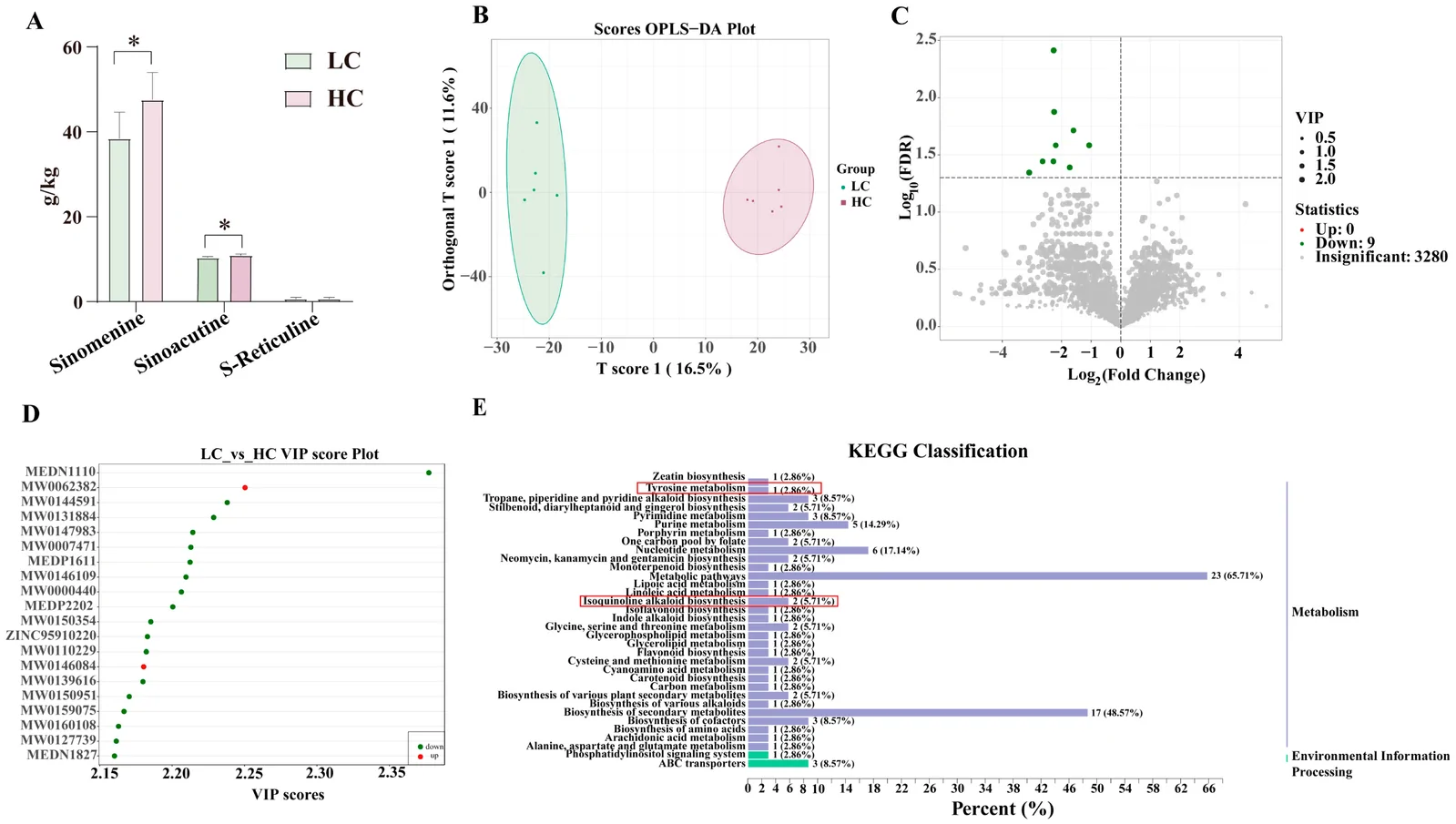
Quantification of major isoquinoline alkaloids and metabolomic analysis of *S. acutum* root. (**A**) Contents of major isoquinoline alkaloids determined by HPLC. Values were presented as mean ± SD (*n* = 6 per group); differences between the HC and LC groups were assessed by one-way ANOVA with Tukey’s test (* *p* < 0.05); note that sinomenine content was the grouping variable, and its comparison is shown to illustrate the group definition. (**B**) OPLS-DA score plot illustrating the metabolic separation between HC and LC groups (*n* = 6). (**C**) Volcano plot of differential metabolites demonstrating relative abundance changes and statistical significance between HC and LC groups (*n* = 6). Red dots represent significantly up-regulated metabolites (differential metabolites were defined as VIP ≥ 1, FDR-adjusted q < 0.05, |log_2_(fold change)| ≥ 1). The dashed lines in (**C**) indicate the screening thresholds for differential metabolites (fold change = 2 or 0.5 and FDR = 0.05). (**D**) VIP ranking plots of the top 20 differentially expressed metabolites from the OPLS-DA model; red indicates higher abundance and green indicates lower abundance in the HC group (direction of change only). (**E**) KEGG classification analysis of differential metabolites; the vertical axis shows the KEGG pathway names, and the horizontal axis shows the number of metabolites annotated to each pathway and their percentage of the total annotated metabolites.

**Figure 2:**
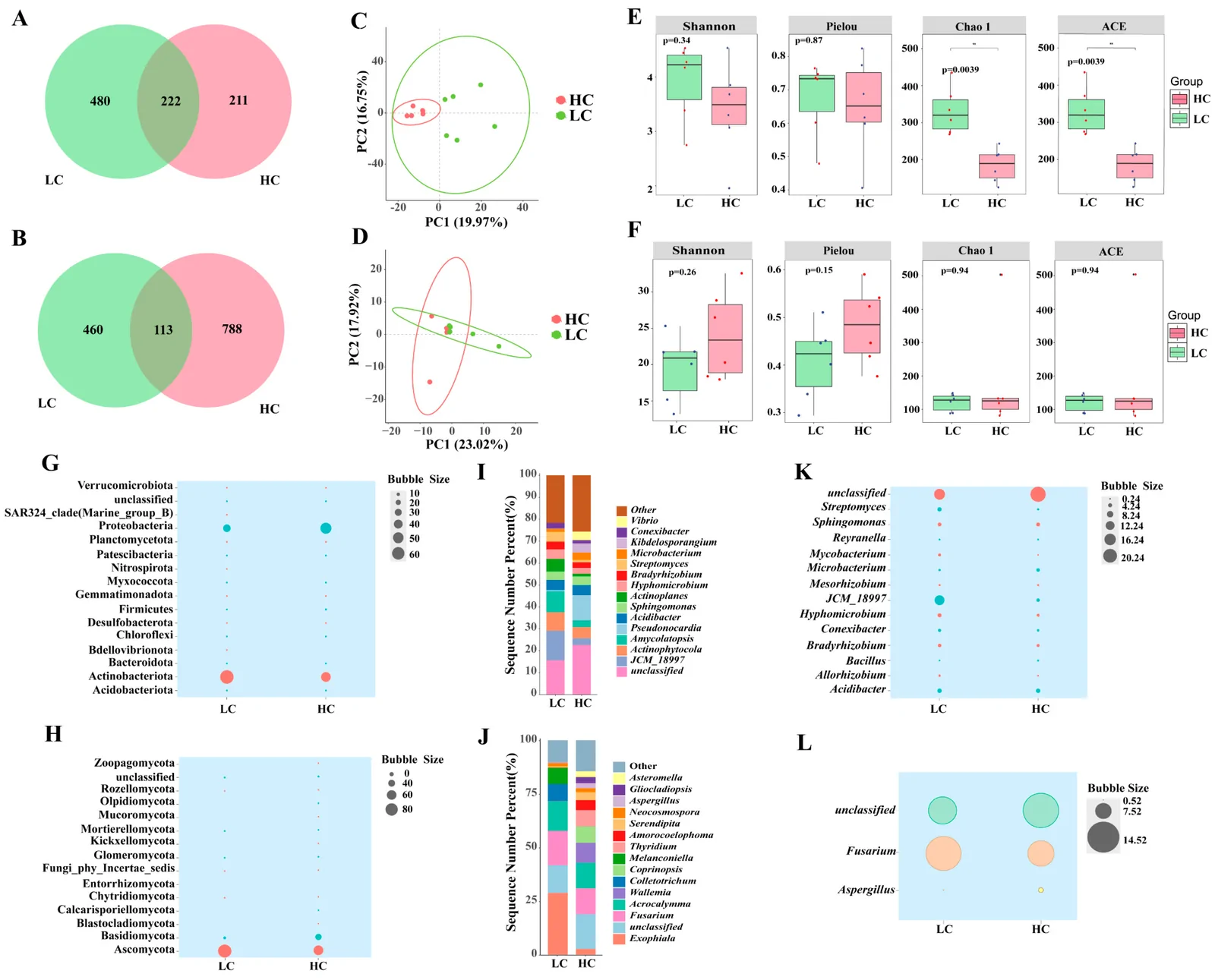
Composition, diversity, and structure of root endophyte communities. (**A**) Venn diagram of shared and unique bacterial ASVs between the HC and LC groups (*n* = 6). (**B**) Venn diagram of shared and unique fungal ASVs between the HC and LC groups (*n* = 6). (**C**) PCA of bacterial community structure; axis percentages indicate the proportion of variance explained by PC1 and PC2. (**D**) PCA of fungal community structure. (**E**) Shannon diversity, Pielou’s evenness, Chao1 and ACE indices of the bacterial community. (**F**) Shannon diversity, Pielou’s evenness, Chao1 and ACE indices of the fungal community. In (**E**,**F**), dots represent the values of individual biological replicates (*n* = 6 per group); ** in (**E**) indicates *p* < 0.01. (**G**) Abundance of bacterial communities at the phylum level; (**H**) Abundance of fungal communities at the phylum level; (**I**) Genus-level composition of bacterial communities. (**J**) Genus-level composition of fungal communities. (**K**) Core bacterial genera analysis. (**L**) Core fungal genera analysis.

Major isoquinoline alkaloids (sinomenine, sinoacutine, and (*S*)-reticuline) were quantified by HPLC in root samples from the 12 individual plants (calibration curves in Figure S1). Based on the pre-defined HC and LC groups (Section 2.1), the HC group exhibited significantly greater sinomenine and sinoacutine contents compared with the LC group (*p* < 0.05, Figure 1A). Since sinomenine content was used to define the HC and LC groups, the intergroup comparison of sinomenine is presented to illustrate the grouping rather than as an independent statistical test; the validity of the grouping was independently supported by PCA of both the metabolomic and microbiome datasets (Figure 1B and Figure 2C,D). In contrast, sinoacutine and (*S*)-reticuline were not used for grouping, and their intergroup comparisons therefore constitute independent tests.

Non-targeted metabolomics analysis of HC and LC roots was performed to investigate metabolic differences. OPLS-DA results demonstrated a clear separation of all samples into two distinct clusters, indicating pronounced metabolic divergence between the HC and LC groups (Figure 1B). Permutation testing gave a significant Q^2^ (*p* < 0.05) but a nonsignificant permuted R^2^Y (*p* = 0.12; Figure S2). Given the small sample size and the high feature-to-sample ratio, the OPLS-DA model is likely to be overfitted, and it is therefore used here only to visualize group separation rather than as confirmatory evidence [29]. Accordingly, VIP values were used only to rank metabolites for visualization (Figure 1D); differential-metabolite calling relied on FDR-adjusted *t*-tests together with the fold-change threshold, and all key alkaloid-related conclusions were independently verified by HPLC quantification, targeted LC-MS/MS, and field inoculation. Metabolites were annotated by database matching, and annotation confidence was assigned following the MSI criteria (Section 2.3). A total of 671 metabolites were annotated, categorized as follows: 37 alkaloids, 278 amino acids and their derivatives, 20 flavonoids, 3 fatty acyls, 11 lipids, 14 nucleotides and their derivatives, 19 alcohols and amines, 74 benzene derivatives, 56 organic acids, 11 phenolic acids, 12 terpenes, 4 glycerolipids and 22 glycerophospholipids, 15 lignans and coumarins, 2 quinones, 3 steroids, 21 heterocyclic compounds and 69 other species.

The volcano plot (Figure 1C) revealed that 9 metabolites were significantly up-regulated in the HC group, including 4 amino acids/derivatives (e.g., Tyr-Trp), 2 alkaloids, 2 benzene derivatives, and 1 alcohol/amine; no metabolite was significantly down-regulated. Further analysis focused on the top 20 metabolites ranked by VIP value from the OPLS-DA model (Figure 1D). Among these top-20 metabolites, 18 were more abundant in the HC group (8 amino acids/derivatives, including H-Tyr-Tyr-Tyr-OH; 4 benzene derivatives; 1 alkaloid; 1 organic acid; 1 lipid; 1 alcohol/amine; 1 lignan/coumarin; and 1 other metabolite) and 2 were less abundant (1 amino acid/derivative and 1 glycerophospholipid). Note that the colors in Figure 1D indicate the direction of change and do not by themselves denote statistical significance. KEGG classification of the differential metabolites (VIP ≥ 1 and nominal *p* < 0.05) (Figure 1E) showed primary enrichment in “Metabolic pathways” and “Biosynthesis of secondary metabolites”. Notably, “Tyrosine metabolism” and “Isoquinoline alkaloid biosynthesis” were significantly represented.

### 3.2. Overview of Endophyte Communities in the Roots of S. acutum

After quality filtering and denoising, a total of 913 bacterial ASVs and 1361 fungal ASVs were identified across all samples. The Venn diagrams (Figure 2A) showed that the bacterial communities shared 222 core ASVs between the HC and LC groups, while the HC and LC groups contained 211 and 480 unique ASVs, respectively. For fungal communities (Figure 2B), 113 core ASVs were shared, with 788 and 460 unique ASVs in the HC and LC groups, respectively. PCA indicated a clear separation between HC and LC groups based on bacterial community structure (Figure 2C; PC1 = 19.97%, PC2 = 16.75%), consistent with low similarity between the groups. The fungal community structures showed partial separation, yet the confidence ellipses overlapped, indicating relatively similar compositions between the two sample groups (Figure 2D, PC1 = 16.08%, PC2 = 14.87%). Alpha diversity analysis (Figure 2E,F) showed no significant differences in the bacterial Shannon index or Pielou’s evenness index between the HC and LC groups, whereas both the bacterial Chao1 and ACE indices were significantly higher in the LC group than in the HC group (*p* < 0.05), indicating greater bacterial richness in the LC group. In contrast, no significant differences were observed in fungal Shannon, Pielou’s evenness, Chao1, or ACE indices between the two groups (*p* > 0.05).

At the phylum level, Acidobacteriota, Actinobacteriota, and Proteobacteria were the dominant bacterial phyla, while Ascomycota and Basidiomycota were the dominant fungal phyla in both groups (Figure 2G,H). Genus-level relative abundance histograms revealed further compositional distinctions (Figure 2I,J). The dominant root endophytic bacterial genera were *JCM-18997*, *Actinophytocola*, *Amycolatopsis*, and *Pseudonocardia*, and the dominant root endophytic fungal genera were *Exophiala*, *Fusarium*, and *Acrocalymma*.

For the core genera analysis (Figure 2K,L), a total of 14 core bacterial genera were identified in the two groups (Figure 2K). Among them, *Microbacterium* and *Bacillus* were consistently present in all samples, highlighting their status as stable core endophytic bacteria. Unclassified bacterial lineages showed the highest relative abundance in both HC and LC, followed by *JCM-18997*, *Acidibacter* and *Sphingomonas*. For the fungal community, three core genera were identified (Figure 2L): unclassified fungi, *Fusarium* and *Aspergillus.* Unclassified fungi and *Fusarium* were the most abundant taxa, whereas *Aspergillus* was present at relatively low abundance.

### 3.3. Isolation and Identification of Endophytic Microorganisms from S. acutum

**Figure 3:**
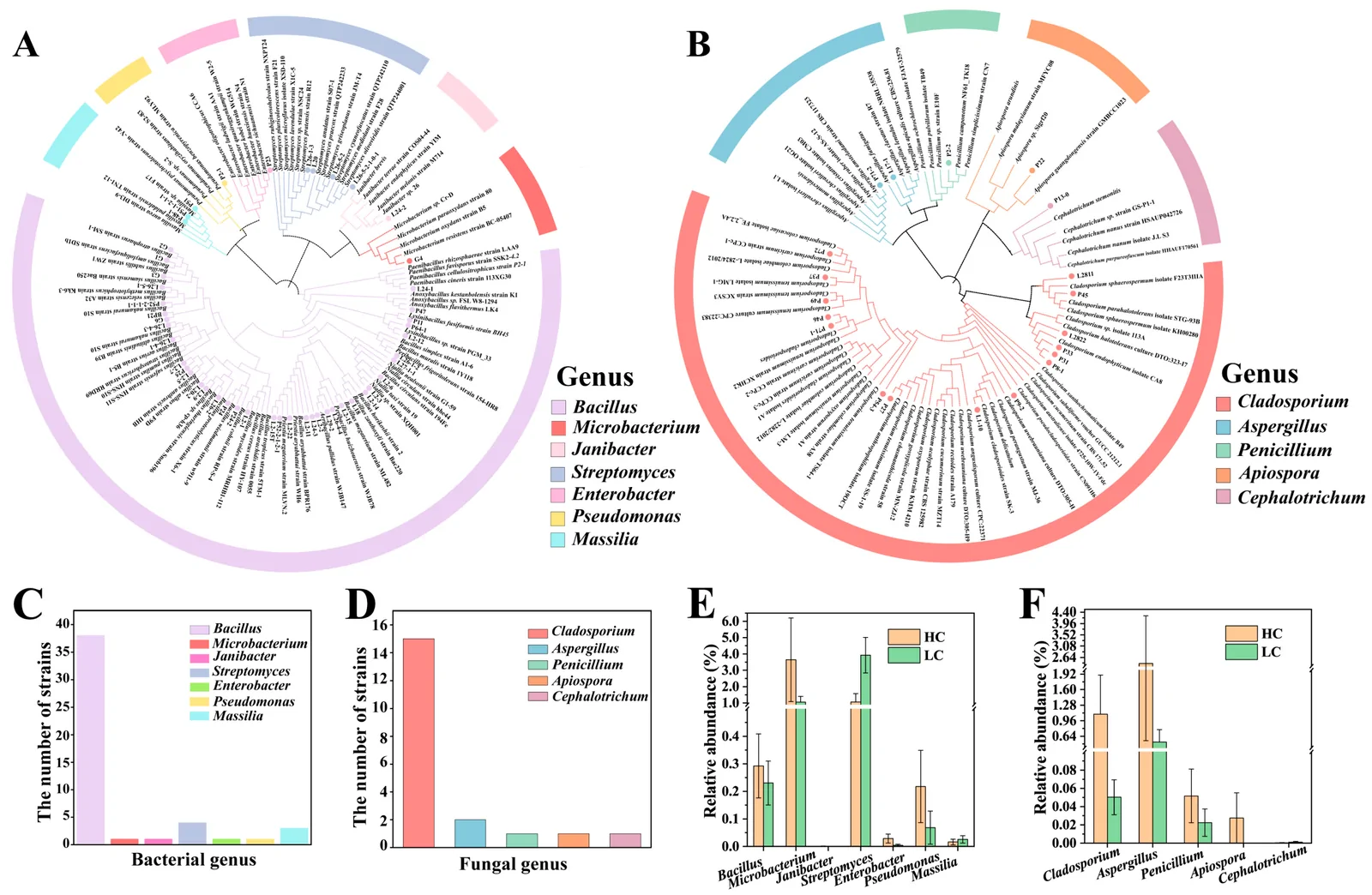
Analysis of isolated endophytic strains. (**A**) Phylogenetic tree of endophytic bacterial isolates based on 16S rRNA sequences (Neighbor-Joining, 1000 bootstrap replicates). (**B**) Phylogenetic tree of endophytic fungal isolates based on ITS sequences. (**C**) Numbers of isolated strains in different bacterial genera. (**D**) Numbers of isolated strains in different fungal genera. (**E**) Relative abundance of the isolated bacterial genera in the amplicon dataset. (**F**) Relative abundance of the isolated fungal genera in the amplicon dataset.

A total of 69 strains, including 49 bacterial strains and 20 fungal strains, were isolated from *S. acutum* roots. Macromorphological characteristics of representative isolated strains are presented in Figure S3A,B. Molecular characterization based on 16S rRNA (bacteria) and ITS (fungi) sequencing and phylogenetic analysis (Figure 3A,B) revealed that the bacterial isolates were assigned to 7 genera (*Bacillus*, *Microbacterium*, *Janibacter*, *Streptomyces*, *Enterobacter*, *Pseudomonas*, and *Massilia*). The fungal strains belonged to 5 genera (*Cladosporium*, *Aspergillus*, *Penicillium*, *Apiospora*, and *Cephalotrichum*). Among the bacterial isolates, *Bacillus* was the most prevalent genus, accounting for 78% of all bacterial isolates (Figure 3C). Among the fungal isolates, *Cladosporium* was the most prevalent, constituting 75% of the fungal isolates (Figure 3D). Analysis of the relative abundance of ASVs belonging to the same genera as the isolated endophytic bacteria and fungi (Figure 3E,F) further confirmed the endophytic nature of these strains.

### 3.4. Correlations Between Endophytic Microorganisms and Key Metabolites

**Figure 4:**
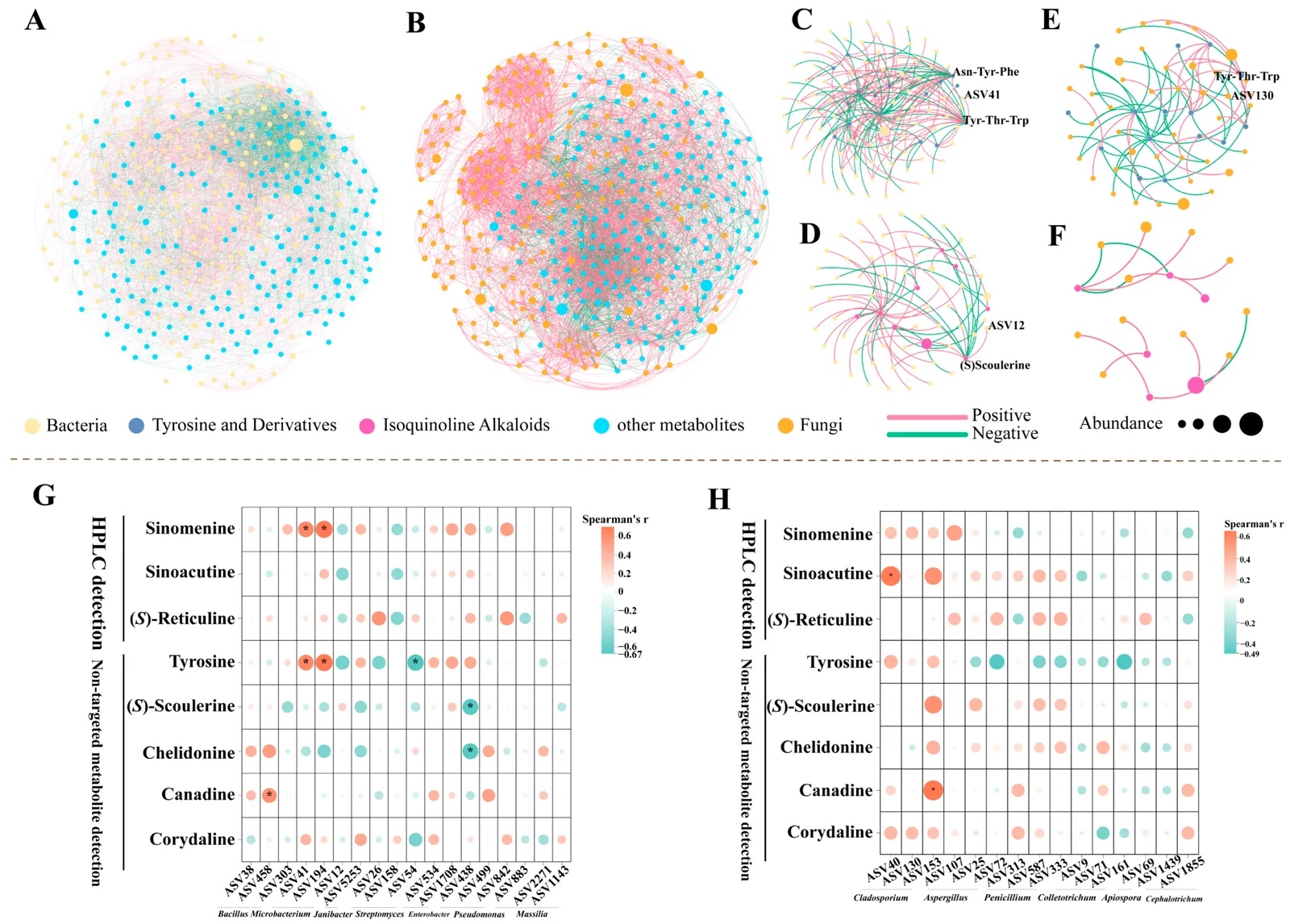
Microbiome-metabolite correlation networks and heatmaps. (**A**) Co-occurrence network of significant Spearman correlations between endophytic bacterial ASVs and root metabolites (|R| > 0.6, q < 0.05). Nodes represent bacterial ASVs and metabolites; edges indicate positive (red) or negative (green) correlations. (**B**) Co-occurrence network of significant Spearman correlations between endophytic fungal ASVs and root metabolites (|R| > 0.6, q < 0.05). Nodes represent fungal ASVs and metabolites; edges indicate positive (red) or negative (green) correlations. (**C**) Subnetwork derived from (**A**) highlighting relationships between L-tyrosine derivatives and endophytic bacterial ASVs. (**D**) Subnetwork derived from (**A**) highlighting relationships between isoquinoline alkaloids and endophytic bacterial ASVs. (**E**) Subnetwork derived from (**B**) highlighting relationships between L-tyrosine derivatives and endophytic fungal ASVs. (**F**) Subnetwork derived from (**B**) highlighting relationships between isoquinoline alkaloids and endophytic fungal ASVs. (**G**) Spearman’s correlation heatmap between bacterial ASVs, total L-tyrosine derivatives, and isoquinoline alkaloids. (**H**) Spearman’s correlation heatmap between fungal ASVs, total L-tyrosine derivatives, and isoquinoline alkaloids. * indicates a significant Spearman correlation (*p* < 0.05).

Co-occurrence networks were constructed between ASVs of the isolated endophytic bacterial/fungal genera and untargeted metabolite features, as shown in Figure 4A,B, with topological parameters detailed in Table S1. Subnetwork analysis focusing on L-tyrosine derivatives and isoquinoline alkaloids (Figure 4C,F) identified several significant positive associations. In the bacterial subnetwork (Figure 4C,D), *Microbacterium* sp. ASV41 was significantly correlated with Tyr-Thr-Trp and Asn-Tyr-Phe, and *Microbacterium* sp. ASV12 was positively associated with (*S*)-scoulerine, an isoquinoline alkaloid. In the fungal subnetwork (Figure 4E,F), ASV130 (classified as *Cladosporium* sp.) showed a significant positive correlation with Tyr-Thr-Trp.

Spearman’s correlation analysis further quantified relationships between the relative abundances of key ASVs (belonging to the genera of isolated strains) and the contents of L-tyrosine derivatives and isoquinoline alkaloids (Figure 4G,H). Among the bacterial taxa, the relative abundances of *Microbacterium* sp. ASV41 and ASV194 displayed significant positive correlations with total L-tyrosine derivatives and sinomenine content. Among the fungal taxa, the relative abundances of *Cladosporium* sp. ASV40 and ASV153 exhibited significant positive correlations with sinoacutine and canadine content, respectively (Figure 4H).

To verify that the observed co-occurrence patterns were not due to random noise, the network properties were compared with those of 10,000 degree-preserving randomized networks. Random network validation results (Table S2) demonstrated that, compared with random networks, the co-occurrence networks of endogenous bacteria and fungi exhibited higher modularity, average clustering coefficients, and average path lengths. This finding indicated that the co-occurrence networks showed non-random topological organization; we note, however, that such comparisons cannot by themselves establish genuine biological interactions, and the individual ASV–metabolite associations remain hypotheses to be tested experimentally.

### 3.5. Fermentation Culture and Alkaloids-Targeted Analysis of Key Endophytic Microbes

**Figure 5:**
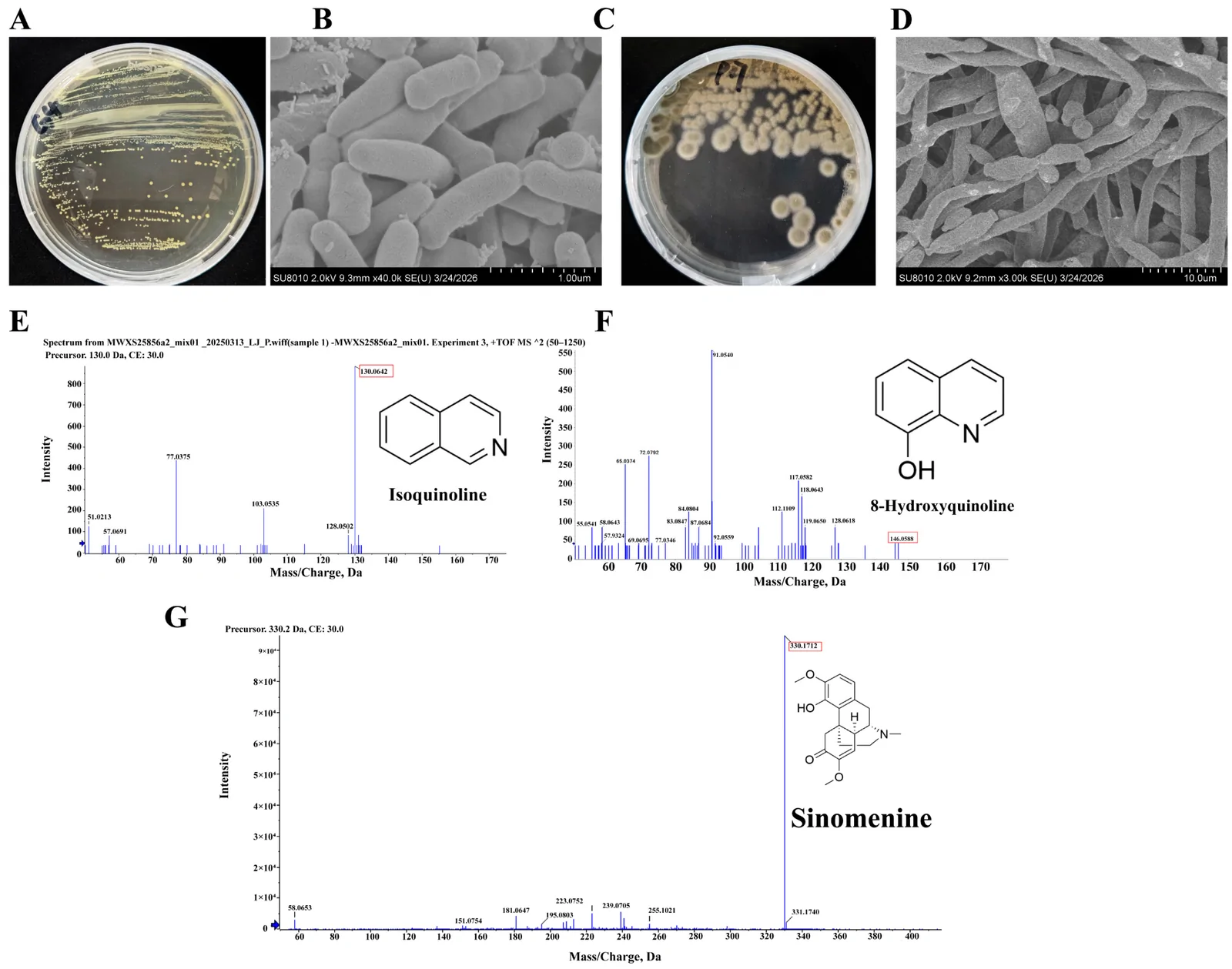
Morphology of *Microbacterium* sp. G4, *Cladosporium* sp. P37 and MS/MS spectra of the targeted metabolites. (**A**) Colony morphology of *Microbacterium* sp. G4. (**B**) SEM micrograph of G4 cells, scale bar = 1 µm. (**C**) Colony morphology of *Cladosporium* sp. P37. (**D**) SEM micrograph of P37 mycelia, scale bar = 10 µm. (**E**) MS/MS spectrum of isoquinoline (tentatively annotated in SG4 cultures; level 2 confidence). (**F**) MS/MS spectrum of 8-hydroxyquinoline. (**G**) MS/MS spectrum of sinomenine (tentatively annotated in P37 cultures; level 2 confidence). Red boxes in (**E**–**G**) indicate the precursor ions (m/z 130.0642, 146.0588, and 330.1712, respectively).

Spearman’s correlation analysis was used to prioritize strains for fermentation screening, and two key strains, *Microbacterium* sp. G4 (Figure 5A,B) and *Cladosporium* sp. P37 (Figure 5C,D), were selected for subsequent fermentation and targeted metabolomics analysis. Under SEM, G4 cells were rod-shaped (bacilli) with smooth and intact cell surfaces, occurring singly or in irregular clusters (Figure 5B, scale bar = 1 µm). SEM observation of strain P37 revealed a dense network of interwoven, septate hyphae with smooth surfaces. The hyphae were uniform in width and branched extensively to form a compact mycelial mat (Figure 5D, scale bar = 10 µm). After removing metabolites detected in the uninoculated blank control, the results (Figure S4 and Table S3) showed that two alkaloids were tentatively annotated in the FG4 group (matching score ≥ 0.5): 8-hydroxyquinoline (Figure 5F) and 7,8-dihydropterin. Blank subtraction indicates that these signals did not originate from the uninoculated medium; however, it does not by itself establish de novo microbial biosynthesis, which would require stable-isotope incorporation experiments (e.g., cultivation with ^13^C_6_-L-tyrosine). In the SG4 group, the same two alkaloids were also tentatively annotated, together with 2-acetamido-3-(1H-imidazol-4-yl)propanoic acid and isoquinoline (Figure 5E). For *Cladosporium* sp. P37 cultured in PDB medium, a total of 164 alkaloids were detected in the fermentation products, including 5 isoquinoline alkaloids, among which sinomenine (Figure 5G) was tentatively annotated.

To further evaluate the reliability of these annotations, the extracted ion chromatograms and MS/MS fragmentation patterns of the annotated isoquinoline alkaloids (isoquinoline from G4, sinomenine from P37) were compared with reference spectra in our in-house library (Figure S5 and Table S4). No authentic standards were co-analyzed, and neither same-run chromatographic overlays, co-injection experiments, nor separation tests for potential isomers were performed; both compounds are therefore reported as putatively annotated (MSI level 2), and isomeric interference cannot be excluded. Both metabolites were detected in all three biological replicates. These results indicated that isoquinoline was tentatively annotated in *Microbacterium* sp. G4 cultures and sinomenine in *Cladosporium* sp. P37 cultures.

### 3.6. Changes in Root Alkaloid Accumulation After G4 Spraying or Irrigation Treatments

**Figure 6:**
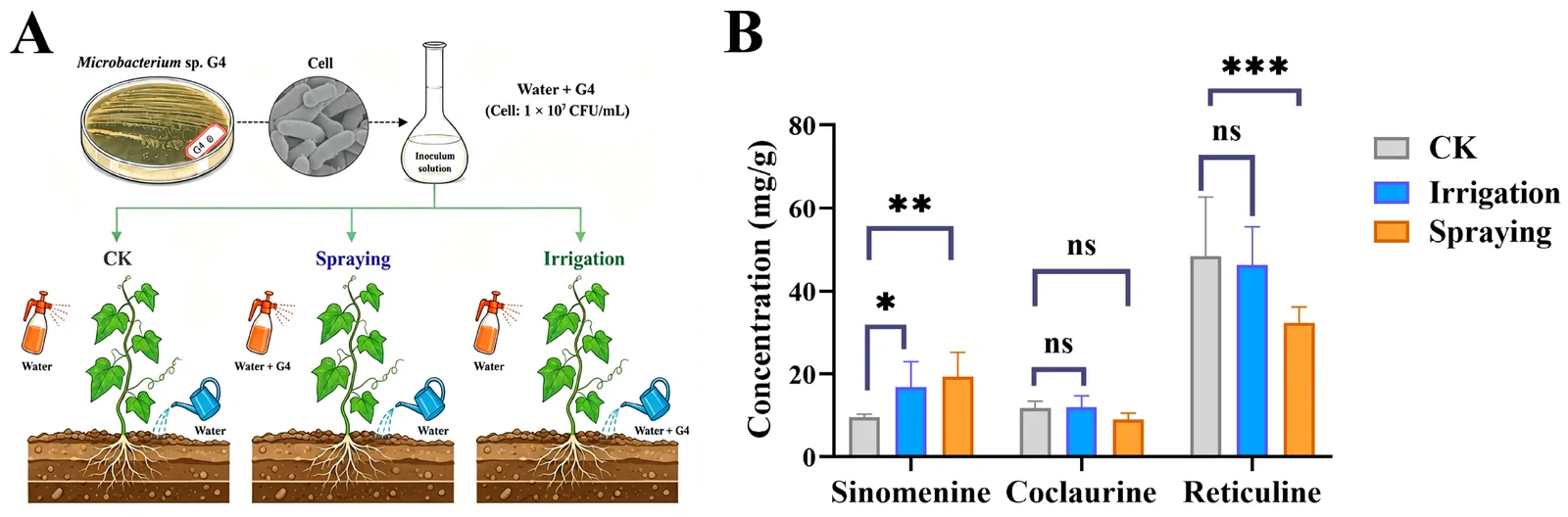
Effects of *Microbacterium* sp. G4 spraying and irrigation on root isoquinoline alkaloid accumulation in *S. acutum*. (**A**) Schematic diagram of the G4 inoculation experiment. The root-derived endophytic bacterium G4 was cultured, prepared as an inoculum suspension (1 × 10^7^ CFU/mL), and applied to *S. acutum* plants by foliar spraying or root irrigation, with water-treated plants serving as the control (CK). (**B**) Contents of representative isoquinoline alkaloids in roots after six months of treatment. Data are presented as mean ± SD (*n* = 6 plants per group); groups were compared by one-way ANOVA followed by Tukey’s multiple-comparison test; ns indicates no significant difference; * *p* < 0.05, ** *p* < 0.01, and *** *p* < 0.001.

To validate the functional role of key endophytic strains, *Microbacterium* sp. G4 was applied to *S. acutum* via foliar spraying and root irrigation, with sterile water serving as the control (CK). Each treatment included 6 biological replicates (*n* = 6 per group), and the experiment lasted 6 months (Figure 6A). As shown in Figure 6B, both the spraying and irrigation treatments significantly increased sinomenine content compared with CK (spraying, *p* < 0.01; irrigation, *p* < 0.05). (*S*)-Reticuline content was significantly lower in the spraying group than in CK (*p* < 0.001), whereas the irrigation group did not differ significantly from CK (*p* > 0.05). (*S*)-coclaurine content showed no significant differences among the three groups (*p* > 0.05). These results demonstrate that *Microbacterium* sp. G4 enhances sinomenine accumulation in *S. acutum* roots; the concomitant decrease in (*S*)-reticuline—the branch-point precursor of the morphinan pathway—in the spraying group is consistent with enhanced metabolic flux toward sinomenine. Foliar spraying was the more effective application method under the tested conditions. It should be noted that colonization of G4 in the treated plants was not verified in this study; the observed effects therefore cannot be attributed with certainty to the establishment of G4 in internal tissues and may also involve phyllosphere- or rhizosphere-associated or indirect systemic effects.

## 4. Discussion

*S. acutum* is a valuable medicinal plant whose pharmacological activities are primarily attributed to isoquinoline alkaloids such as sinomenine [30,31]. Endophytic microorganisms are widely recognized as important components of plant-associated microbiomes, and they can influence host secondary metabolism through complex metabolic and ecological interactions [32,33]. In this study, we combined metabolomics, amplicon sequencing, targeted isolation, fermentation experiments, and field validation to explore the association between root endophytic communities and alkaloid accumulation in *S. acutum*. Rather than simply reflecting taxonomic composition, the observed microbial patterns suggested that host metabolic status was closely linked to endophytic community assembly and functional potential.

The differences between HC and LC plants indicated that alkaloid-rich roots harbored a distinct and potentially more interactive bacterial community. Although no significant differences were detected in bacterial Shannon diversity or Pielou’s evenness between the two groups, the LC group exhibited significantly higher bacterial richness (Chao1 and ACE indices; *p* < 0.05). This pattern suggests that higher alkaloid content may exert stronger selective pressure on bacterial colonization, favoring a community with lower species richness but potentially tighter functional coordination [34]. Such a trade-off between diversity and interaction complexity has also been reported in other plant-associated microbiomes, where specialized metabolic environments promoted stronger ecological filtering and tighter microbial co-occurrence patterns [32,35,36]. In contrast, fungal α-diversity did not differ significantly between the two groups, implying that fungi may be less sensitive than bacteria to alkaloid-associated host metabolic changes in *S. acutum*. Similar phylum- or genus-level stability has been observed in some medicinal plant systems, suggesting that bacterial and fungal endophytes may respond differently to host chemical environments [37]. The enrichment of *Microbacterium* and *Bacillus* as core bacterial genera in both HC and LC plants further supported the idea of a stable functional backbone in the root microbiome. Members of these genera have been reported to possess broad ecological adaptability, plant colonization capacity, and metabolic versatility, which may explain their persistence across host chemotypes [38,39]. Overall, the microbiome differences between HC and LC plants may have been shaped by host-derived chemical selection, niche specialization, and intermicrobial cooperation, rather than by taxonomic richness alone.

The capacity of endophytes to produce host-related metabolites has been increasingly demonstrated in medicinal plants. Endophytic bacteria and fungi can synthesize bioactive compounds identical or structurally similar to those of their hosts, and such metabolites may arise through pathway complementation or independent evolution of analogous biosynthetic routes [40,41]. In this context, the putative detection of isoquinoline in cultures of *Microbacterium* sp. G4 and of sinomenine in cultures of *Cladosporium* sp. P37 was particularly noteworthy. In plants, benzylisoquinoline alkaloid biosynthesis begins with the condensation of two L-tyrosine derivatives, dopamine and 4-hydroxyphenylacetaldehyde, to form (*S*)-norcoclaurine, which is converted through (*S*)-coclaurine, (*S*)-N-methylcoclaurine and (*S*)-reticuline into the morphinan branch, ultimately yielding sinomenine via sinoacutine [9,18,42]. The two lines of evidence concerning G4 should not be interpreted as contradictory. Isoquinoline, tentatively annotated in G4 cultures, is a simple aromatic heterocycle and is not a biosynthetic intermediate of sinomenine, a morphinan-type alkaloid whose formation from (*S*)-reticuline requires multiple enzymatic steps that have so far been documented only in plants [9,42]. It is therefore unlikely that G4 synthesizes sinomenine de novo. Rather, the in vitro annotation suggests that G4 possesses nitrogen-heterocycle metabolism that may contribute to precursor supply. This interpretation is consistent with the organ-specific model of sinomenine metabolism in *S. acutum*—precursor synthesis in leaves, modification in stems, and accumulation in roots [18]—under which leaf-applied G4 would act as a precursor supplier rather than as a producer of the end-product alkaloid. The significant positive correlations between *Microbacterium* ASVs and total L-tyrosine derivatives, together with the enrichment of tyrosine-derived metabolites in HC plants [18,43], were consistent with a possible precursor-provisioning role, which nonetheless remains speculative pending direct evidence such as isotope-tracing experiments.

Our data are associative (ASV–metabolite correlations) or endpoint-based (metabolite annotation in fermentation cultures and alkaloid quantification after field inoculation); they therefore cannot localize the step(s) at which G4 or P37 might act, whether through precursor supply, modulation of specific host enzymatic steps, or indirect physiological effects. A caveat of the correlation analysis is that the HC/LC groups were defined by sinomenine content: any ASV differing in abundance between the groups will inevitably correlate with sinomenine, because group membership drives both variables—a form of circular inference analogous to “double dipping” in systems neuroscience [44]. The correlation networks are therefore hypothesis-generating rather than confirmatory, and the observed ASV–metabolite associations may reflect group-level differences rather than direct pairwise biological interactions. Association-based prioritization was consequently used only to select candidate strains, which were then examined in independent fermentation and field inoculation experiments. Although the metabolite profiles differed depending on the culture medium, these findings indicate the presence of alkaloid-related metabolites in the cultures but do not demonstrate de novo microbial biosynthesis [45]. Similar examples have been reported in other medicinal plant systems, where endophytes were able to produce paclitaxel-like compounds, vinblastine-related alkaloids, or other host-associated metabolites under specific culture conditions [46]. Our findings therefore added to the growing evidence that endophytes from medicinal plants are associated with pharmaceutically relevant alkaloid metabolism. In addition, the detection of 8-hydroxyquinoline is of interest because this compound has been reported to exhibit antimicrobial activity, while isoquinoline and its derivatives are recognized natural products with diverse biological activities [47,48]. Thus, the metabolic repertoire of these isolates suggested both biosynthetic novelty and potential pharmaceutical relevance.

Endophytes may enhance the accumulation of medicinal compounds in host plants through direct and indirect mechanisms. In our field validation, inoculation with *Microbacterium* sp. G4 increased the root content of sinomenine while decreasing (*S*)-reticuline, indicating that this strain could positively modulate host alkaloid accumulation, possibly by promoting metabolic flux through the morphinan branch. Consistent with this finding, root irrigation with *Cladosporium* sp. P37 significantly increased root sinomenine content in a parallel field trial reported in our companion study [18], suggesting that both endophytes can modulate host alkaloid accumulation, albeit with different application preferences. Such effects are consistent with previous studies showing that endophytes can improve plant secondary metabolite production by supplying precursors, altering phytohormone balance, and activating defense-related signaling [43]. Because L-tyrosine is a major precursor for isoquinoline alkaloid biosynthesis, the enrichment of tyrosine-derived metabolites in HC plants may reflect a metabolic state favorable for alkaloid formation. It is plausible that endophyte-driven changes in precursor availability or metabolic flux contributed to this phenotype, although the precise mechanism remains to be determined. Similar microbe-mediated enhancement of active metabolites has been reported in other medicinal plants, where inoculation with beneficial endophytes or plant growth-promoting microbes increased the accumulation of terpenoids, phenolic compounds, or alkaloids [49,50]. These studies, together with our results, suggest that endophytes could be developed as microbial resources for improving the medicinal quality of cultivated plants. The observation that foliar spraying was more effective than root irrigation for G4 also indicated that the application route may strongly influence colonization efficiency and functional performance, which should be considered in future inoculation studies. Notably, our previous multi-omics study revealed an organ-specific pattern of isoquinoline alkaloid metabolism in S. acutum, in which precursors are synthesized in the leaves, modified in the stems, and accumulated in the roots [18]. This pattern offers a plausible explanation for the effect of foliar G4 application: leaf-associated G4 may enhance the supply of alkaloid precursors—such as tyrosine-derived intermediates—in the leaves, which are subsequently transported to and accumulated in the roots. Several additional, non-exclusive mechanisms are also conceivable. Some bacterial endophytes can enter aerial tissues and translocate within the plant to reach stems and roots [51], although colonization was not verified here. Foliar inoculation may also act through systemic signaling, as beneficial microbes can prime phytohormone- and defense-related signaling that systemically modulates metabolism in distal organs [52], and jasmonate signaling is a well-established regulator of alkaloid biosynthesis [53]. Furthermore, part of the foliar inoculum inevitably reaches the soil surface during spraying, so a rhizosphere-mediated contribution cannot be excluded, and inoculation-induced changes in root exudation could indirectly reshape the root-associated community and its metabolic output [34,35]. Distinguishing among these possibilities will require colonization tracking combined with tissue-specific transcriptomic and metabolite profiling.

The present study has several limitations. Although the correlations, fermentation assays, and field experiments collectively supported a functional association between endophytes and alkaloid accumulation, they did not fully establish the in planta enzymatic mechanisms; the small sample size (*n* = 12, six plants per group) limits statistical power, and the metabolomic and microbiome comparisons should be regarded as hypothesis-generating. Moreover, for megavariate datasets in which the number of variables greatly exceeds the number of subjects, validation tools such as cross-validation and permutation tests become unreliable, and model outcomes depend strongly on the specific samples used [29]. Genome sequencing and transcriptomic analyses of G4 and P37 will be necessary to identify biosynthetic gene clusters and regulatory elements involved in metabolite production. In addition, multi-site and multi-year field trials will be required to evaluate the consistency of the inoculation effects under different environmental conditions, and colonization of the inoculated strain was not demonstrated; future studies should track G4 by re-isolation, strain-specific qPCR, or fluorescent tagging to confirm establishment in planta. In particular, stable-isotope labeling (e.g., ^13^C_6_-L-tyrosine tracing) will be required to test whether the annotated alkaloids are synthesized de novo by the isolates. Finally, future studies employing targeted and absolutely quantitative analyses will be necessary to further establish the biological relevance of these associations.

## 5. Conclusions

In summary, this study revealed significant differences in endophytic community structure and metabolite profiles between *S. acutum* groups with high and low sinomenine content. *Microbacterium* and *Bacillus* were identified as core endophytic bacterial genera. Correlation analysis suggested potential associations between isoquinoline alkaloids and both *Microbacterium* and *Cladosporium*, while L-tyrosine and its derivatives were also correlated with *Microbacterium*. In vitro fermentation experiments indicated that isoquinoline was putatively annotated in cultures of *Microbacterium* sp. G4 when the strain was cultivated on Czapek-Dox medium supplemented with L-tyrosine, and that sinomenine was putatively annotated in cultures of *Cladosporium* sp. P37 grown in PDB medium. These results provide preliminary evidence for the association of these endophytes with alkaloid-related metabolites. Field validation further showed that foliar spraying of G4 significantly increased the sinomenine content (*p* < 0.01) but decreased the (*S*)-reticuline content (*p* < 0.001) in roots; root irrigation of G4 also increased sinomenine (*p* < 0.05), though to a lesser extent. Collectively, these correlative and experimental lines of evidence provide initial support for the hypothesis that specific endophytic microorganisms can contribute to isoquinoline alkaloid accumulation in *S. acutum*. Although these findings remain exploratory, they lay the groundwork for future studies on endophyte–alkaloid interactions in medicinal plants.

## Data Availability

The raw amplicon sequencing data have been deposited in the NCBI SRA database under accession numbers PRJNA1287093 and PRJNA1287097.

## Supplementary Materials

The following supporting information can be downloaded at: https://www.mdpi.com/article/10.3390/plants15203211/s1, Figure S1: Calibration curves for quantitative detection; Figure S2: Permutation test results validating the OPLS-DA model; Figure S3: Morphological characterization of the isolated endophytic strains; Figure S4: MS/MS spectra of alkaloids tentatively annotated in cultures of strains G4 and P37; Figure S5: Reference MS/MS spectra from the in-house database; Table S1: Topological parameters of the co-occurrence network among endophytic bacteria/fungi and metabolites in *S. acutum*; Table S2: Parameters employed for the random network validation of interactions between endophytic microbes and metabolites in *S. acutum*; Table S3: Targeted annotation of alkaloids in cultures of *Microbacterium* sp. G4 and *Cladosporium* sp. P37; Table S4: MS/MS fragmentation information of isoquinoline and sinomenine from the in-house reference database.
