# Supplementary material for: Microbiome-Associated Regulation of Isoquinoline Alkaloids in Sinomenium acutum Reveals Functional Endophytes for Sinomenine Accumulation
Source: Plants (Basel). 2026 Oct 19;15(20):3211. doi: 10.3390/plants15203211 (PMC13708848; doi:10.3390/plants15203211)
Supplement: Supplementary file 1 [file plants-15-03211-s001.zip › plants-4526087-supplementary.pdf]

Supplementary data

Supplementary material methods

1. The details of DNA extraction and Illumina HiSeq sequencing

The V5-V7 variable region of bacterial 16S rRNA gene was amplified by primers 799F (5'-AACMGGATTAGATACCCKG-3') and 1193R (5'-ACGTCATCCCCACCTTCC-3'). The internal transcribed spacer (ITS) region of fungi was amplified with primers ITS1-1F-F (5'-CTTGGTCATTTAGAGGAAGTAA-3') and ITS1-1F-R (5'-GCTGCGTTCTTCATCGATGC-3'). The PCR reaction conditions were as follows: an initial denaturation at 98°C for 1 min, 30 cycles of 10 s denaturation at 98°C, 30 s for annealing at 50°C, and 30 s for elongation at 72°C, and a final extension at 72°C for 5 min.

2. Specific detection methods for UPLC-MS/MS

Detection based on the UPLC-MS/MS detection platform and self-built database. The sample extracts were analyzed using a UPLC-ESI-MS/MS system (UPLC, ExionLC™ AD, SCIEX) coupled to a triple quadrupole-linear ion trap mass spectrometer (QTRAP 4500, SCIEX).

The analytical conditions were as follows. UPLC: column, Agilent SB-C18 (1.8 µm, 2.1 mm * 100 mm); The mobile phase consisted of solvent A, pure water with 0.1% formic acid, and solvent B, acetonitrile with 0.1% formic acid. Sample measurements were performed with a gradient program that employed the starting conditions of 95% A, 5% B. Within 9 min, a linear gradient to 5% A, 95% B was programmed, and a composition of 5% A, 95% B was kept for 1 min. Subsequently, a composition of 95% A, 5.0% B was adjusted within 1.1 min and kept for 2.9 min. The flow rate was 0.35 mL/min; The

column oven was set to 40°C; The injection volume was 2 μL. The effluent was alternatively connected to an ESI-triple quadrupole-linear ion trap (QTRAP)-MS.

The ESI-QTRAP-MS/MS source operation parameters were as follows: source temperature 500°C; ion spray voltage (IS) 5500 V (positive ion mode)/-4500 V (negative ion mode); ion source gas I (GSI), gas II (GSII), curtain gas (CUR) were set at 50, 60, and 25 psi, respectively; the collision-activated dissociation (CAD) gas (nitrogen) was set to medium. QQQ scans were acquired as MRM experiments. The declustering potential (DP) and collision energy (CE) of the individual MRM transitions were further optimized. A specific set of MRM transitions were monitored for each period according to the metabolites eluted within this period.

Figure S1. Calibration curves for quantitative detection. (A) Sinomenine; (B) Sinoacutine; (C) Reticuline.

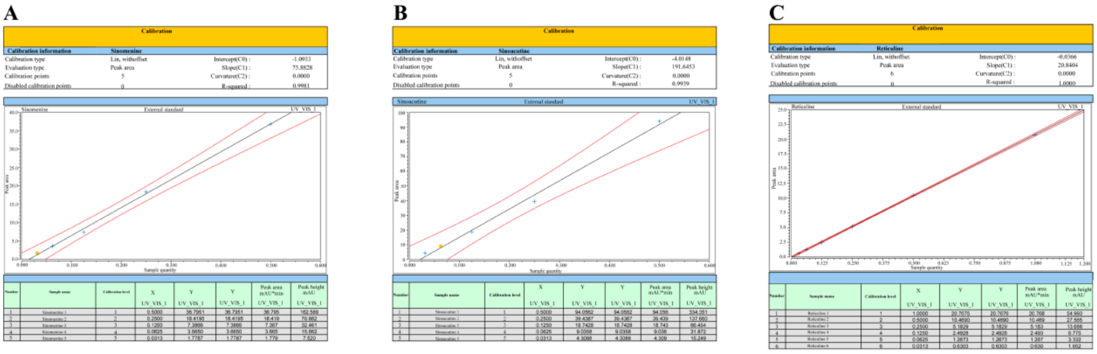

Figure S2. Permutation test results validating the OPLS-DA model.

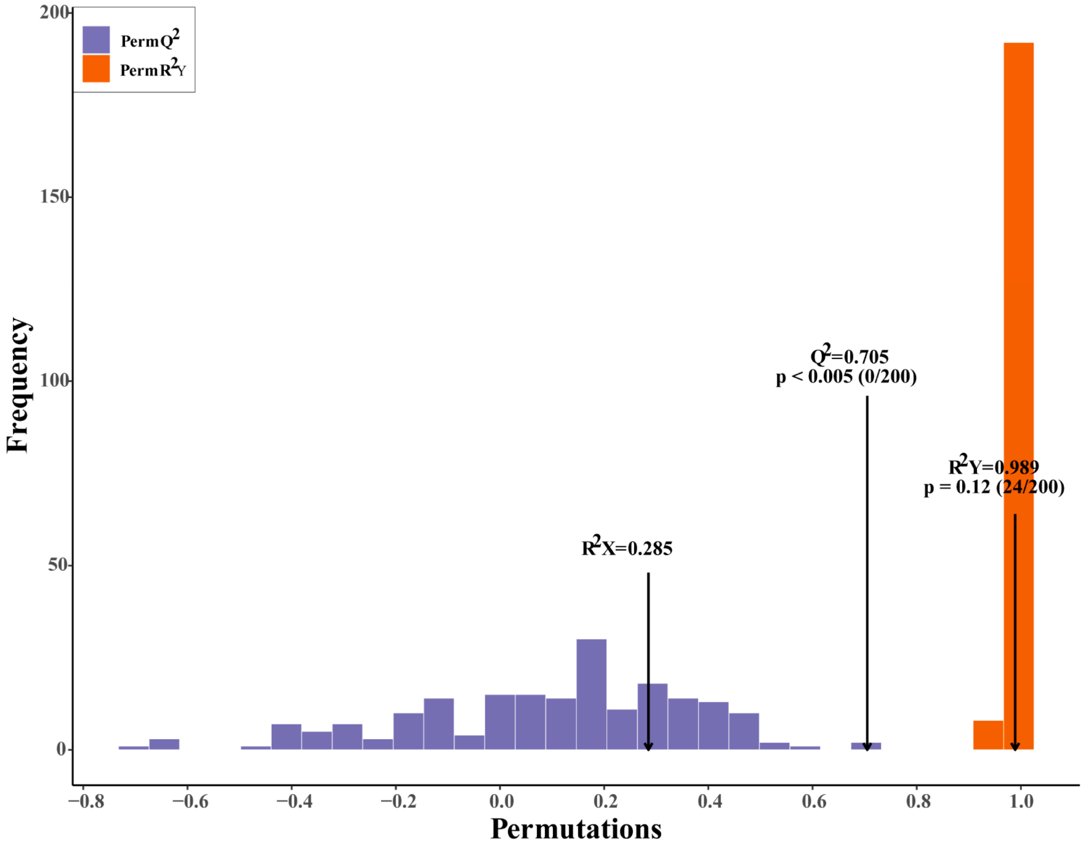

Figure S3. Morphological characterization of the isolated endophytic strains. (A) Colony morphology of the isolated bacterial strains cultured on LB agar plates at 30°C for 2-3 days. (B) Colony morphology of the isolated fungal strains cultured on PDA plates at 25°C for 7-14 days.

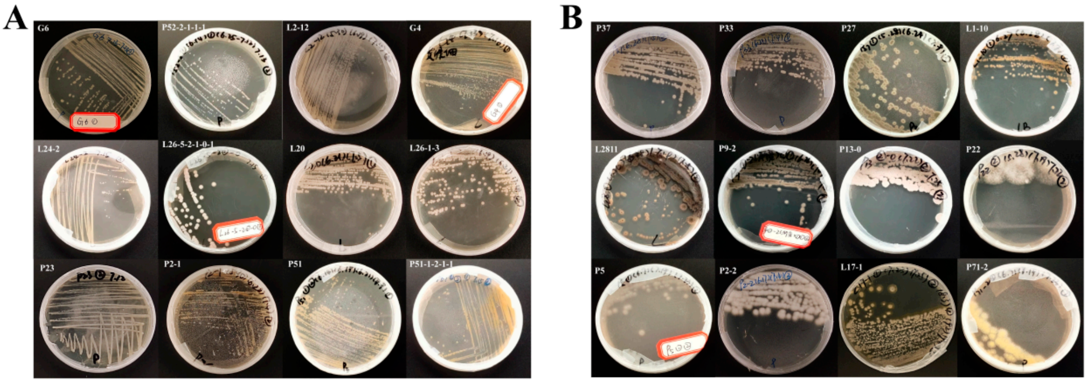

Figure S4. MS/MS spectra of alkaloids tentatively annotated in cultures of strains G4 and P37. Tandem mass spectra showing the fragmentation patterns of alkaloid compounds detected in strains G4 and P37. Metabolite annotation was based on the concordance of retention time (RT) and MS/MS fragmentation patterns (Q1/Q3 MRM transitions) with an in-house database; the RT tolerance was set at 0.2 min. Because a unit-resolution triple-quadrupole instrument was used, no accurate-mass information was available. All compounds were annotated without co-analyzed authentic standards and are therefore reported as putatively annotated compounds (MSI level 2). (A) 8-hydroxyquinoline; (B) Isoquinoline; (C) Sinomenine; (D) 7,8-dihydropterin; (E) 2-acetamido-3-(1H-imidazol-4-yl)propanoic acid; (F) 6-acetamido-3-aminohexanoic acid; (G) 2-[(3-hydroxy-4-methoxyphenyl)methyl]-7-methoxy-1-methyl-3,4-dihydro-1H-isoquinolin-6-ol; (H) Sanguinarine; (I) (1R)-7-hydroxy-1-[(3-hydroxy-4-methoxyphenyl)methyl]-6-methoxy-2-methyl-3,4-dihydro-1H-isoquinolin-2-ium-2-olate.

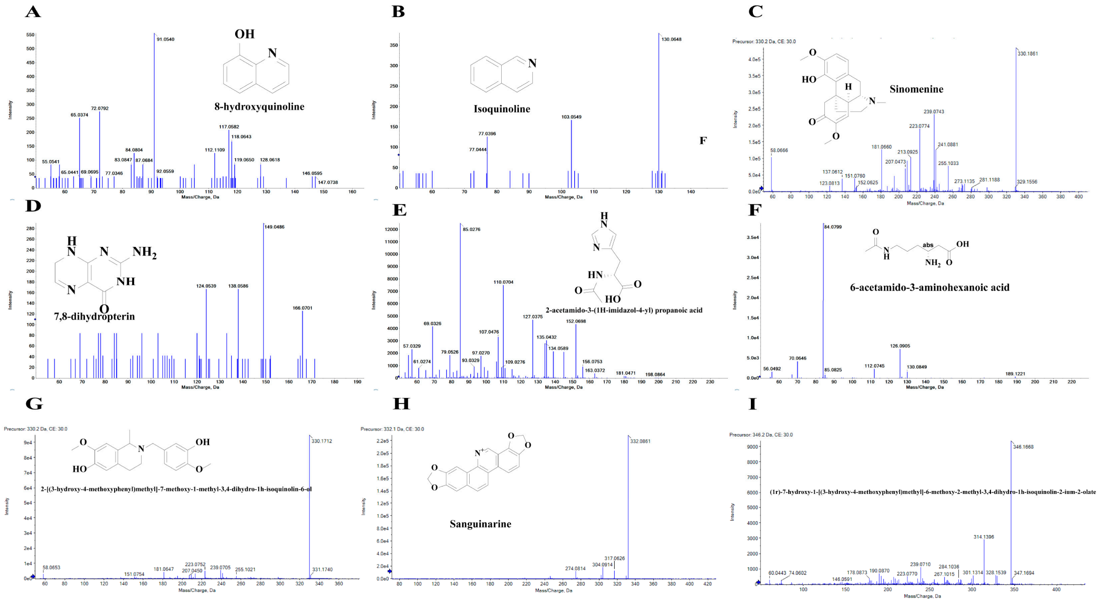

Figure S5. Reference MS/MS spectra from the in-house database. (A) Reference MS/MS spectrum of isoquinoline; (B) reference MS/MS spectrum of sinomenine.

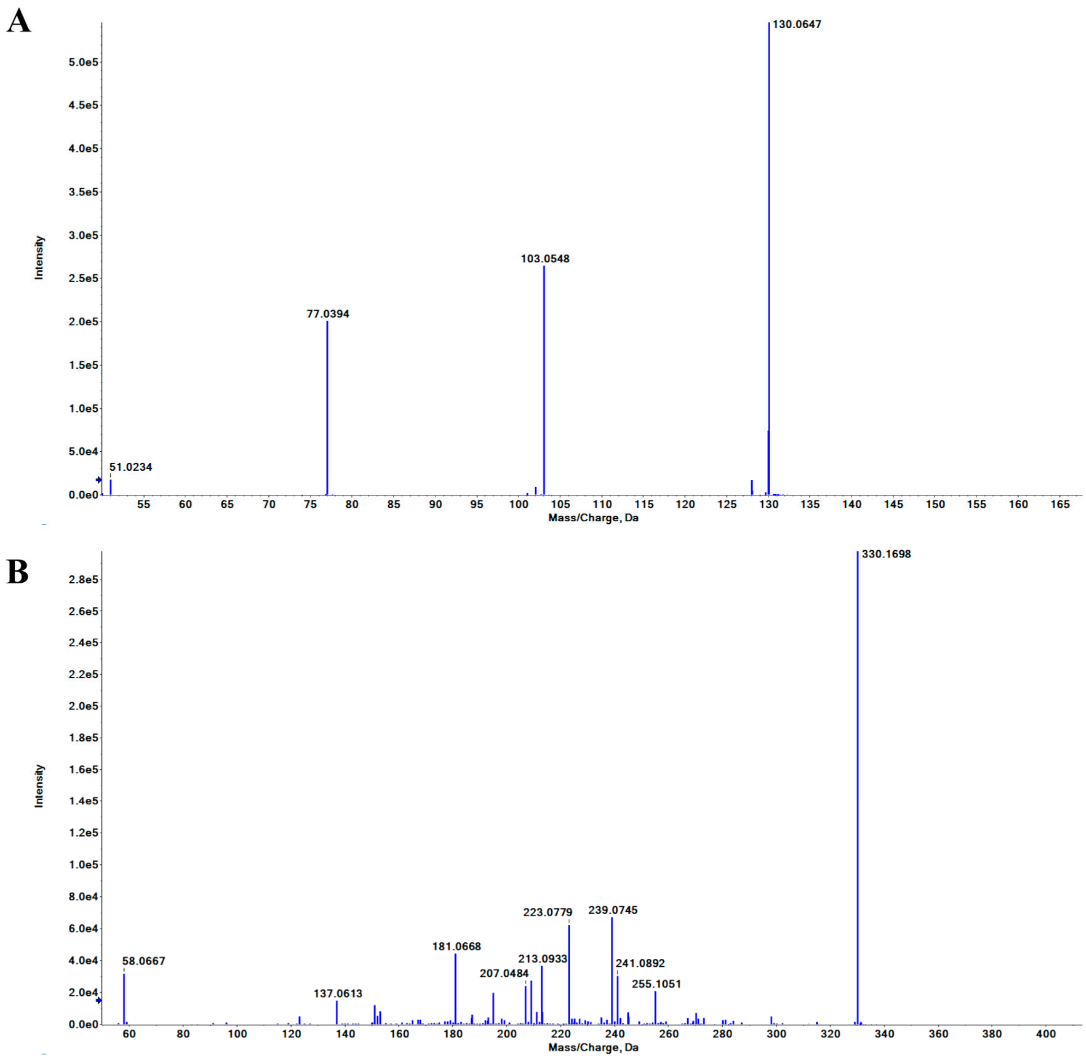

Table S1. Topological parameters of the co-occurrence network among endophytic bacteria/fungi and metabolites in *S. acutum*.

Network Index	Bacteria-metabolism	Fungi-metabolism
Nodes	400	400
Links	5940	4799
Average degree	14.85	11.998
Average weighted degree	14.85	11.998
Network diameter	9	10
Map density	0.037	0.03
Connected components	2	1
Modularity	0.432	0.547
Average clustering coefficient	0.215	0.255
Average path length	2.719	3.035
Positive link	64.04%	71.76%
Negative link	35.96%	28.24%

Table S2. Parameters employed for the random network validation of interactions between endophytic microbes and metabolites in *S. acutum*.

Network Index	Bacteria-metabolism	Fungi-metabolism
Nodes	400	400
Links	5940	4799
n	10000	10000
Map density	0.037±0.000	0.030±0.000
Modularity	0.144±0.003	0.164±0.003
Average clustering coefficient	0.074±0.001	0.060±0.001
Average path length	2.027±0.001	2.162±0.001

Table S3. Targeted annotation of alkaloids in cultures of *Microbacterium sp.* G4 and *Cladosporium sp.* P37.

Compounds	Class II	Level	FG4	SG4	P37	Q1 (Da)	Q3(Da)	RT(min)	DP	CE
8-hydroxyquinoline	Quinoline alkaloids	2	246101	263111	N/A	146.06	91.05	2.38	50	30
Isoquinoline	Isoquinoline alkaloids	2	N/A	17650	59976	130.06	103.05	2.7	50	30
Sinomenine	Isoquinoline alkaloids	2	N/A	N/A	386188.4	330.17	181.07	2.68	20	40
7,8-dihydropterin	Alkaloids	2	99074	805507	N/A	166.07	149.05	1.49	50	30
2-acetamido-3-(1H-imidazol-4-yl)propanoic acid	Alkaloids	2	N/A	14219	N/A	198.09	110.07	0.92	50	30
6-acetamido-3-aminohexanoic acid	Alkaloids	2	N/A	N/A	43643	189.12	70.07	0.87	50	30
2-[(3-hydroxy-4-methoxyphenyl)methyl]-7-methoxy-1-methyl-3,4-dihydro-1h-isoquinolin-6-ol	Isoquinoline alkaloids	2	N/A	N/A	8163666	330.17	239.07	2.54	50	30
Sanguinarine	Isoquinoline alkaloids	2	N/A	N/A	132474	332.09	304.09	4.86	50	30
(1r)-7-hydroxy-1-[(3-hydroxy-4-methoxyphenyl)methyl]-6-methoxy-2-methyl-3,4-dihydro-1h-isoquinolin-2-ium-2-olate	Isoquinoline alkaloids	2	N/A	N/A	170623	346.16	192.1	2.62	50	30

Note: Annotation confidence was assigned according to the Metabolomics Standards Initiative (MSI) criteria described in Section 2.3 of the main text. All compounds were annotated by matching retention time and MS/MS fragmentation patterns (Q1/Q3 MRM transitions) against an in-house database without co-analyzed authentic standards and are therefore reported as putatively annotated compounds (MSI level 2). The values in the table represent the relative peak areas of each compound, with larger areas indicating higher analyte concentrations.

Table S4. MS/MS fragmentation information of isoquinoline and sinomenine from the in-house reference database.

Compounds	MS2
Isoquinoline	130；103；77；51
Sinomenine	239；223；181；241；213；209
